# Anthrax Toxin Receptor Drives Protective Antigen Oligomerization and Stabilizes the Heptameric and Octameric Oligomer by a Similar Mechanism

**DOI:** 10.1371/journal.pone.0013888

**Published:** 2010-11-08

**Authors:** Alexander F. Kintzer, Harry J. Sterling, Iok I. Tang, Evan R. Williams, Bryan A. Krantz

**Affiliations:** 1 Department of Chemistry, University of California, Berkeley, California, United States of America; 2 California Institute for Quantitative Biomedical Research (QB3), University of California, Berkeley, California, United States of America; 3 Department of Molecular and Cell Biology, University of California, Berkeley, California, United States of America; Griffith University, Australia

## Abstract

**Background:**

Anthrax toxin is comprised of protective antigen (PA), lethal factor (LF), and edema factor (EF). These proteins are individually nontoxic; however, when PA assembles with LF and EF, it produces lethal toxin and edema toxin, respectively. Assembly occurs either on cell surfaces or in plasma. In each milieu, PA assembles into a mixture of heptameric and octameric complexes that bind LF and EF. While octameric PA is the predominant form identified in plasma under physiological conditions (pH 7.4, 37°C), heptameric PA is more prevalent on cell surfaces. The difference between these two environments is that the anthrax toxin receptor (ANTXR) binds to PA on cell surfaces. It is known that the extracellular ANTXR domain serves to stabilize toxin complexes containing the PA heptamer by preventing premature PA channel formation—a process that inactivates the toxin. The role of ANTXR in PA oligomerization and in the stabilization of toxin complexes containing octameric PA are not understood.

**Methodology:**

Using a fluorescence assembly assay, we show that the extracellular ANTXR domain drives PA oligomerization. Moreover, a dimeric ANTXR construct increases the extent of and accelerates the rate of PA assembly relative to a monomeric ANTXR construct. Mass spectrometry analysis shows that heptameric and octameric PA oligomers bind a full stoichiometric complement of ANTXR domains. Electron microscopy and circular dichroism studies reveal that the two different PA oligomers are equally stabilized by ANTXR interactions.

**Conclusions:**

We propose that PA oligomerization is driven by dimeric ANTXR complexes on cell surfaces. Through their interaction with the ANTXR, toxin complexes containing heptameric and octameric PA oligomers are similarly stabilized. Considering both the relative instability of the PA heptamer and extracellular assembly pathway identified in plasma, we propose a means to regulate the development of toxin gradients around sites of infection during anthrax pathogenesis.

## Introduction

Anthrax toxin (Atx) [1] is a key virulence factor produced by pathogenic strains of *Bacillus anthracis*. Atx consists of three nontoxic protein components: protective antigen (PA) is an 83-kDa, cell-binding component of Atx that ultimately forms an oligomeric translocase channel, which delivers the two enzyme components, lethal factor (LF) and edema factor (EF), into the cytosol of a host cell [2], [3], [4]. LF is a 90-kDa, zinc-dependent protease [5], [6], [7], which cleaves host-cell mitogen-activated protein kinase kinases [5], [6]. While PA and LF are individually nontoxic, the combination of LF and PA creates lethal toxin (LT), which can alter cellular physiology and cause death [8]. EF is a 89-kDa, Ca^2+^/calmodulin-activated adenylyl cyclase [9], [10], [11]. Analogously, PA and EF combine to form edema toxin (ET), which induces tissue swelling and may also cause death [8], [12].

To achieve cytotoxicity, PA, LF, and EF must first self-assemble into holotoxin complexes. There are two different types of assembly pathways: (i) a cell-surface pathway and (ii) a plasma-based/extracellular pathway. In the former mechanism, PA forms complexes on the surface of host cells in a receptor-dependent manner. PA first binds to one of two known Atx receptors (ANTXR): ANTXR1 [13] and ANTXR2 [14]. The PA-ANTXR interaction [15] is stable and dissociates with a half-life measured in days [16]; the interaction involves domains 2 and 4 in PA, such that the latter domain coordinates the receptor' Ca^2+^ or Mg^2+^ metal ion adhesion site [15], [16], [17], [18]. Receptor-bound PA is then cleaved by a furin-type protease to make the proteolytically-activated form, called _n_PA. After a 20-kDa portion of _n_PA (PA_20_) dissociates, the remaining 63-kDa (PA_63_), receptor-bound portion assembles into a mixture of ring-shaped heptameric (PA_7_) [17], [19], [20] and octameric (PA_8_) [21], [22] oligomers. The complexes are endocytosed [23] and brought to an acidic compartment [24]. Under acidic pH conditions, the PA oligomers form translocase channels [25], [26], allowing the passage of LF and EF into the cytosol.

In a second assembly mechanism, PA, LF, and EF form LT and ET complexes in the blood. *In vivo* studies of anthrax infection measured high concentrations of toxin components in the blood of infected animals [2], [3]. At the later stages of anthrax, PA and LF concentrations reach up to 100 µg/mL and 20 µg/mL, respectively [27]. Analysis of the circulating toxin components revealed that the majority of detectable PA exists as the proteolytically-processed PA_63_ form, which is either assembled or capable of assembling with LF in a manner analogous to what is observed on cell surfaces [27], [28], [29]. *In vitro* bovine-plasma assembly experiments reveal that PA oligomers and LT complexes may form efficiently from full-length PA and LF, where the resulting oligomers contain mixtures of PA_7_ and PA_8_ complexes [21], [22]. PA_7_ complexes have a strong propensity for aggregation under physiological conditions (due to their premature conversion to the channel state), suggesting that the toxin requires additional stabilization mechanisms to remain efficacious during infection [21], [22], [30]. Since PA_8_ complexes are more stable in plasma under physiological conditions (pH 7.4, 37°C), it has been proposed [22] that the soluble fraction of LT circulating in bloodstream of infected animals [28] may contain an enriched population of the PA_8_ oligomer.

While it is clear that PA_8_ functions as a stable complex in plasma, it is unknown whether PA_7_ and PA_8_ complexes are stabilized differentially on cell surfaces. When the PA heptamer binds to its cellular receptor, ANTXR, the interaction inhibits channel formation, significantly stabilizing PA complexes by ∼2 pH units [15], [17]. Previous studies have also shown that ANTXR2 dimerization leads to an increase in the formation of PA_8_ in vitro, presumably by populating dimeric intermediates along the assembly pathway [21]. Here we explore the role of the ANTXR in the PA assembly pathway and determine the degree of stabilization the receptor imparts on the two different PA oligomers produced during assembly.

## Results

### PA oligomerization is accelerated in the presence of ANTXR2 dimers

**Figure 1:**
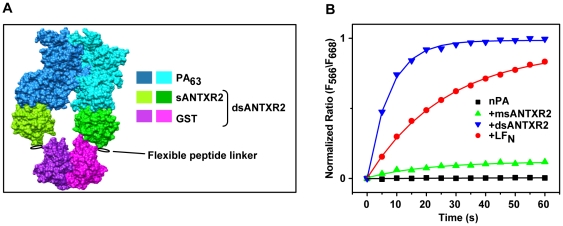
ANTXR2 dimerization stimulates PA assembly. (**A**) A manually constructed model of dsANTXR2 bound to two adjacent PA_63_ subunits in a PA_7_ oligomer. The surface rendering is colored according to the legend on the right. The model is based upon the crystal structures of GST (PDB 5GST[31]) and PA_7_(msANTXR2)_7_ (PDB 1TZN[17]). A flexible linker is shown in black that links the carboxy-terminus of GST to the amino-terminus of msANTXR2. (**B**) FRET-probed PA-assembly kinetics at pH 7.4. A 1∶1 mixture of _n_PA K563C*AF_555_ and _n_PA K563C*AF_647_ monomers (100 nM total monomer) was either allowed to assemble on its own (black ▪) or mixed with 100 nM of the following assembly co-factors, dsANTXR2 (blue ▾), msANTXR2 (green ▴), or LF_N_ (red •), and allowed to assemble. To track the time course of PA assembly, the ratio of acceptor to donor fluorescence (F_668_/F_566_) was measured every five minutes for one hour at room temperature. The resulting records are normalized to the largest signal obtained for the dsANTXR co-assembly reaction. Solid lines are best-fit lines obtained using a second-order rate model (Eq. 1). The rate constants, *k*, are 0.19 (±0.01) s^−1^ for dsANTXR2, 0.031 (±0.003) s^−1^ for LF_N_, and 0.05 (±0.03) s^−1^ for msANTXR2, and the amplitudes, *A*, are −1.12 (±0.02) for dsANTXR2, −1.29 (±0.04) for LF_N_, and −0.15 (±0.02) for msANTXR2. Note due to the lack of an observable change in FRET signal, no kinetic parameters were obtained for the assembly of _n_PA alone, and the data were fit to a straight line.

While ANTXR2 dimerization enhances the formation of PA_8_ [21], it is not known whether the rate and extent of PA oligomerization are influenced by a dimeric ANTXR2 complex (dsANTXR2). A previous study indicates that LF's PA binding domain (LF_N_, the first 263 residues of LF) can increase the rate of PA oligomerization, while soluble monomeric ANTXR2 extracellular domain (msANTXR2) did not appear to influence assembly greatly [16]. To ask whether ANTXR2 dimerization affects the rate of PA oligomerization, we produced a soluble extracellular dsANTXR2 construct, which contains an amino-terminal fusion of glutathione S-transferase (GST) and the extracellular domain of ANTXR2. The GST domain forms tight homodimers [31] with an equilibrium dissociation constant of less than 1 nM [32]. We previously verified that this construct is fully homodimeric by mass spectrometry [21]. Structurally, the amino-termini of adjacent ANTXR2 extracellular domains in the crystal structure of the PA_7_(ANTXR2)_7_ structure [17] are ∼55 Å apart (Fig. 1A). This distance is similar to the distance between the carboxy-termini (∼44 Å) in the crystal structure of the GST dimer [31], and we infer that the 6-amino acid linkers positioned between the GST domains and the ANTXR2 domains can span this 11-Å differential. Finally, as our model in Figure 1A indicates, the amino terminus of the ANTXR2 points away from the PA-ANTXR interface, and there are no steric constraints, which would prevent the ANTXR2 dimer from forming via the GST interaction either in a PA dimer or higher-order PA_7_/PA_8_ oligomer complex. Thus this dimeric fusion construct could in principle stabilize the formation of productive dimeric PA intermediates during assembly.

We measure the rate of PA oligomerization using Förster resonance energy transfer (FRET). AlexaFluor 555 and 647 reactive maleimides are conjugated to PA monomers via sulfhydryl modification of the unique Cys residue introduced by the K563C mutation, forming PA K563C*AF_555_ and PA K563C*AF_647_, respectively. These residues are sufficiently close together in the PA oligomer to allow FRET between adjacent monomers [16]. We monitor PA assembly using a 1∶1 mixture of AF_555_-donor-and AF_647_-acceptor-labeled nicked-PA (_n_PA) monomers (50 nM each) and the ratio of the fluorescence emission intensities at 668 and 566 nm (F_668_/F_566_). As shown in previous studies [16], we also find the extent of _n_PA oligomerization in the absence of co-assembly factors is slight over the time course of one hour (Fig. 1B). Consistent with previous studies [16], the presence of msANTXR2 does not stimulate PA assembly appreciably; rather it modestly increases the extent of assembly when compared to the _n_PA control. We fit the resulting FRET, *F*, vs time (*t*) data with a second-order rate model [16]

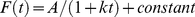
(1)


For msANTXR2 stimulated assembly, we estimate that the observed rate constant, *k*, is 0.05 (±0.03) s^−1^ with an amplitude, *A*, of −0.15 (±0.02). Both LF_N_ and dsANTXR2 greatly stimulate the extent of PA assembly, *A* values of −1.12 (±0.02) and −1.29 (±0.04), respectively (Fig. 1B), where the relative extent of assembly over msANTXR2 stimulated assembly is increased ∼7 and 9-fold, respectively. We find that, relative to msANTXR, dsANTXR2, with a *k* of 0.19 (±0.01) s^−1^, accelerates the rate of oligomerization ∼4-fold (Fig. 1B). Thus we conclude that ANTXR2 dimerization stimulates both the rate and extent of PA assembly.

### Mass spectrometry analysis of PA_7_(LF_N_)_3_ and PA_8_(LF_N_)_4_ co-complexes with msANTXR2

**Figure 2:**
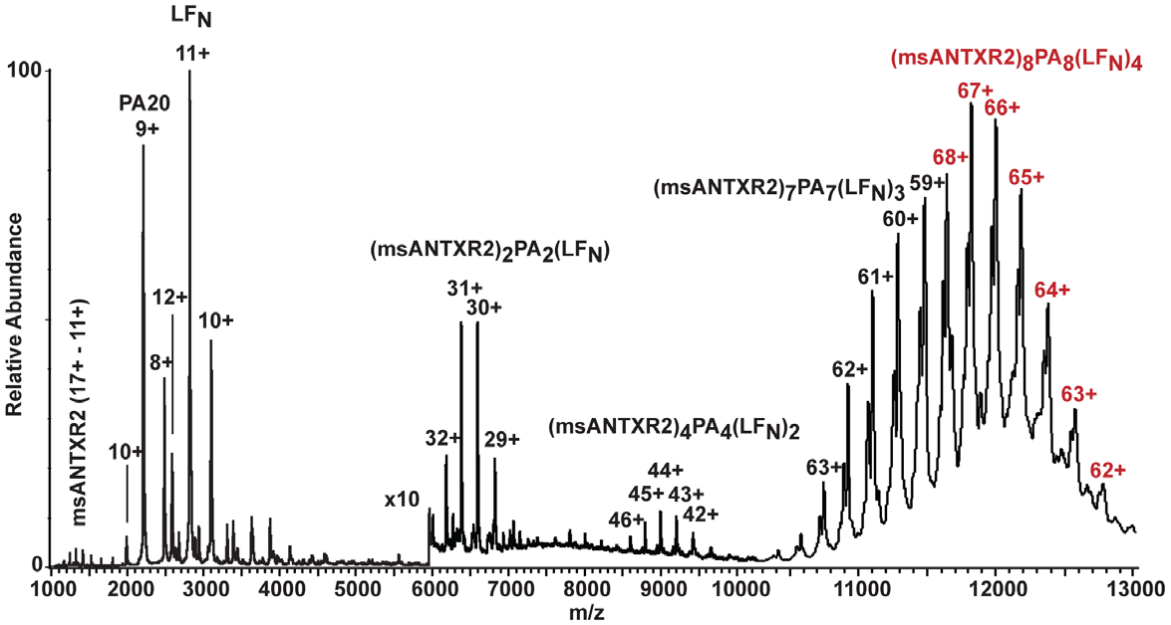
Nanoelectrospray mass spectrometry analysis of PA-LF_N_-msANTXR2 oligomer complexes. Nanoelectrospray MS of sANTXR-PA-LF_N_ complexes (∼2 µM) in 200 mM ammonium acetate, 2 mM ammonium bicarbonate, 0.2 mM magnesium acetate, pH 7.8. The *y*-axis is scaled 10× in the range *m*/*z* 6000–13,000, and the *x*-axis is expanded in the range *m*/*z* 10,000–13,000 to aid viewing low relative abundance and closely spaced peaks in these regions. See also Table 1 for the respective molecular mass values for each complex.

Quantitative fluorescence [16] and X-ray crystallographic studies [17] similarly report that PA_7_ binds 7 msANTXR2 domains. Using mass spectrometry (MS), we can also determine the stoichiometry of anthrax toxin complexes. Previous studies reported that _n_PA and LF_N_ form PA-LF_N_ complexes containing PA_7_ and PA_8_ [21]. Here we find that when this mixture of PA-LF_N_ complexes is liganded by an excess of msANTXR2, two high-molecular mass species of 677,125 (±70) Da and 791,823 (±128) are formed. These masses are consistent with the theoretical molecular masses of the PA_7_(LF_N_)_3_(msANTXR2)_7_ and PA_8_(LF_N_)_4_(msANTXR2)_8_ complexes, respectively (Fig. 2, Table 1). (Note, for simplicity, we refer to these complexes as PA_7_-msANTXR2 and PA_8_-msANTXR2, respectively.) Also present at slightly lower relative abundances are the assembly intermediates, PA_2_LF_N_(msANTXR2)_2_ and PA_4_(LF_N_)_2_(msANTXR2)_4_ (Fig. 2). Finally, the free monomers, msANTXR, PA_20_ and LF_N_, are observed in the range *m*/*z* 1000–3500 (Fig. 2). Thus we conclude that the PA oligomer architecture does not preclude the binding of a complete stoichiometric complement of ANTXR domains.

**Table 1:** Measured^a^ and theoretical^b^ molecular masses for msANTXR-PA-LF_N_ complexes.

Sample	Measured molecular mass^a^ (Da)	Theoretical molecular mass^b^ (Da)	Deviation (%)
msANTXR_8_(PA)_8_(LF_N_)_4_	791,823 (±128)	790,609	0.15
msANTXR_7_(PA)_7_(LF_N_)_3_	677,125 (±70)	676,327	0.12
msANTXR_4_(PA)_4_(LF_N_)_2_	395,722 (±33)	395,305	0.11
msANTXR_2_(PA)_2_LF_N_	197,815 (±19)	197,652	0.08

a Molecular masses are measured using nanoelectrospray MS according to the method described in Kintzer et al. [21].

b Theoretical molecular masses are derived using the amino acid sequences of msANTXR, PA_63_, and LF_N_.

### EM analysis of the stability of PA_7_ and PA_8_ co-complexes with msANTXR2

**Figure 3:**
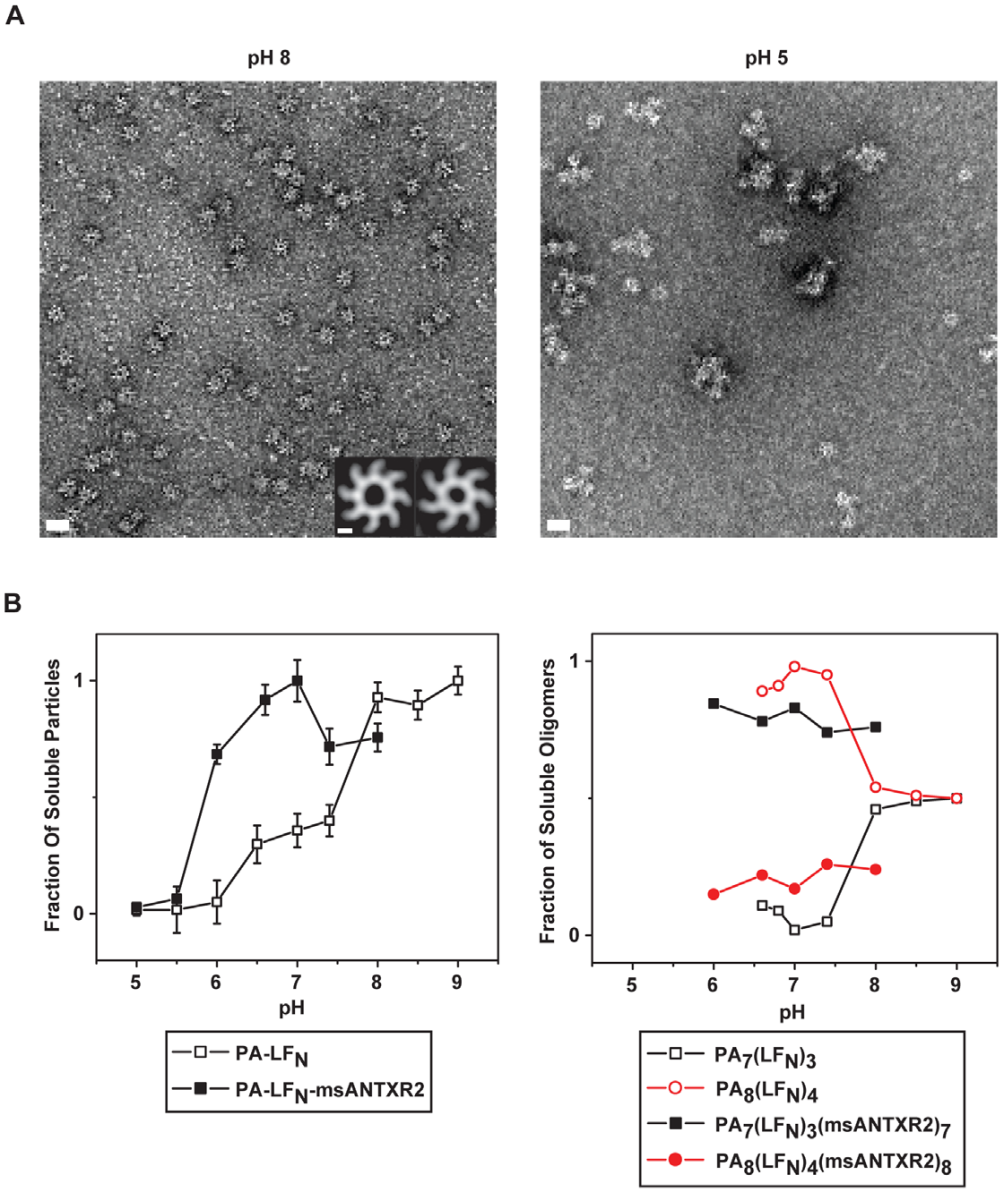
EM analysis of the stability of PA_7_-msANTXR2 and PA_8_-msANTXR2 complexes from pH 8.0 to 5.0. (**A**) Representative micrographs (49,000×) of PA-msANTXR2 complexes following a 5-minute exposure to 37°C at either pH 8.0 (left) or pH 5.0 (right). A 20-nm scale bar is shown in white for either micrograph. (inset on left) Class-average images of PA_7_-msANTXR2 and PA_8_-msANTXR2 complexes; a 5-nm scale bar is shown. (**B**) Quantitative analysis of the number of soluble PA oligomers and the relative proportions of PA_7_ and PA_8_, identified from electron micrographs at each pH. (left) A plot of the average number of soluble prechannels versus pH for both free PA complexes (□, data taken from [22]) and msANTXR2-bound PA complexes (▪) complexes. Error bars are propagated from the standard deviations of the mean number of particles obtained from at least 10 micrographs for each pH. (right) A plot of the relative proportions of PA_7_ (black ▪) and PA_8_ (red •) complexes determined using class-average image analysis for both PA-LF_N_ (open symbols, data taken from [22]) and PA-LF_N_-msANTXR2 (filled symbols) complexes.

Using electron microscopy (EM), we measure the relative pH-dependent stabilities of the two different PA oligomers. Here we equate complex stability with the ability of the complex to remain soluble. Prior studies show that insoluble toxin complexes are also inactive [22]. To examine the stability of these complexes, we briefly incubate the oligomeric mixture of PA-msANTXR2 complexes (78% PA_7_, 22% PA_8_) for 5 min at 37°C under a range of pH conditions, and then we analyze the composition of the resulting soluble complexes by EM (Fig. 3). At pH 8.0, we observe mainly distinct axially-oriented oligomeric particles, but at pH 5.0, we largely observe indiscernible aggregates (Fig. 3A). We find that the number of soluble PA-msANTXR2 prechannel co-complex particles per micrograph decreases as a function of pH with a pH midpoint of 5.8 (Fig. 3B, Table 2). The sharp decrease in the average number of soluble prechannel co-complex particles per micrograph also coincides with the appearance of large aggregates (Fig. 3A). The appearance of the latter is indicative of premature formation of the PA channel [22]. Data from a similar study of ANTXR-free PA oligomer particles [22] show a different result, where the pH-dependent transition was biphasic with two different pH midpoints, corresponding to the PA_7_ and PA_8_ oligomers (Fig. 3B) [22]. We conclude that when PA_7_ and PA_8_ complexes bind to msANTXR2 they are stabilized similarly and their solubility as a function of pH reveals a coincident sigmoidal pH dependence.

**Table 2:** Negative-stain^a^ EM analysis PA-msANTXR2 co-complexes following an exposure at 37°C^b^.

pH	Mean number of particles per micrograph^c^	Oligomeric composition^d^		
		PA_8_ (%)	PA_7_ (%)	Total particles (*N*)
8.0	18 (±4)	23	77	364
7.4	20 (±7)	26	74	381
7.0	28 (±8)	17	83	494
6.5	26 (±6)	22	78	482
6.0	19 (±4)	16	84	857
5.5	2 (±1)	n.d.^e^	n.d.	n.d.
5.0	1 (±1)	n.d.	n.d.	n.d.

a Negative-stain electron micrographs using uranyl acetate stain, 2%.

b A pre-assembled population of PA-msANTXR2 complexes (containing 78% PA_7_ and 22% PA_8_) was incubated for 5 minutes at 37°C at the specified pH.

c The mean number of particles per micrograph (*n* of 10 micrographs) given as ±s.d.

d Oligomeric composition is determined using crystal-structure-referenced alignment and classification analysis [21], [22]. The percentage reported is computed from the total number of particles, *N*, comprising all PA_7_ and PA_8_ classes, where oligomeric composition is equal to the total number of PA_7_ or PA_8_ particles divided by *N*.

e n.d., not determined. Class-average image analyses of these pH conditions are not shown due to the low particle counts observed. The low particle counts are attributed to severe aggregation (as shown in Fig. 3A).

We then asked if the monophasic sigmoidal transition in the number of soluble PA-msANTXR2 prechannel co-complex particles resulted from the two different PA oligomers having identical pH dependencies for channel formation (and aggregation). To address this question, we measured the relative proportions of the PA_7_-msANTXR2 and PA_8_-msANTXR2 complexes at each pH. From the negative-stain electron micrographs taken at each pH value, we apply a reference-based alignment, classification, and averaging analysis of all distinct soluble particles (Fig. 3A, inset) [21]. This analysis, which generates average images of the ring-shaped particles, shows that the percentages of PA_7_ and PA_8_ are constant over the range of pH 8 to 6 (Fig. 3B, Table 2). The constant percentages observed for these msANTXR2-bound oligomer complexes are in stark contrast to what is observed with msANTXR-free PA oligomer complexes (Fig. 3B). Thus, when in complex with the msANTXR2 domain, PA_7_ and PA_8_ have identical pH dependent stabilities and tend to aggregate with identical pH midpoints of 5.8.

### Circular dichroism changes in PA_7_-msANTXR2 and PA_8_-msANTXR2 complexes

**Figure 4:**
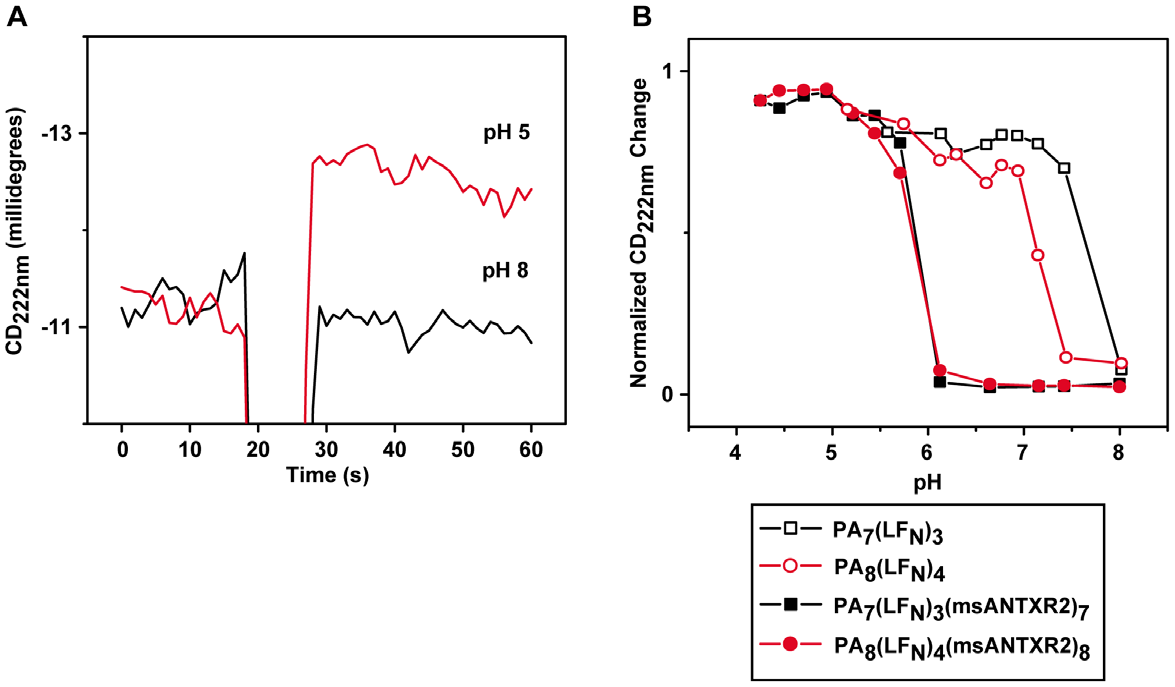
The pH dependence of CD-signal changes for PA_7_- and PA_8_-msANTXR2 complexes. (**A**) Time-course records of the CD signal at 222 nm (CD_222_) for either an acid pulse (pH 5.0 final, red trace) or a control with no pH pulse (pH 8.0 final, black trace). (**B**) The pH-dependence of the CD_222_-signal change for PA_7_(LF_N_)_3_ (black □, data taken from [22]), PA_8_(LF_N_)_4_ (red ○, data taken from [22]), PA_7_(LF_N_)_3_(msANTXR2)_7_ (black ▪), PA_8_(LF_N_)_3_(msANTXR2)_8_ (red •) complexes. Traces were normalized to the initial and final CD_222_ signals obtained.

Circular dichroism (CD) spectroscopy studies provide a structural probe for PA's transition from the prechannel state to the channel state. This pH-dependent structural transition likely explains the decrease in complex stability and solubility observed, since PA channels tend to aggregate in solution [22]. The secondary structure increases reported by CD signals have been associated with channel formation because the pH-dependent CD-signal change occurs at similar pH values as observed with other probes for channel formation, including EM, MS, and SDS-PAGE [22]. Using the CD signal at 222 nm (CD_222nm_), we can measure the pH-dependent conformational changes in purified PA_7_-msANTXR2 and PA_8_-msANTXR2 complexes (Fig. 4A, B). These purified samples also contain full complements of LF_N_, but they are highly enriched >90% in either the PA_7_ or PA_8_ oligomer [21]. Records of the pH-dependent CD_222nm_ signal change of the PA oligomer-msANTXR2 complexes at 37°C (Fig. 4A) show equivalent results for either PA oligomer from pH 4.0 to 8.0 (Fig. 4B). The pH-dependent CD_222nm_ signal changes for the PA_7_-msANTXR2 and PA_8_-msANTXR2 complexes coincide, such that the prechannel-to-channel transition midpoint is pH 5.8 (Fig. 4B). Relative to the PA oligomers assayed under similar conditions in the absence of msANTXR [22], this pH 5.8 midpoint is stabilized by ∼1.8 and ∼1.2 pH units for the PA_7_ and PA_8_ oligomers, respectively.

### The pH-dependence of PA_7_-msANTXR2 channel formation is temperature-independent

**Figure 5:**
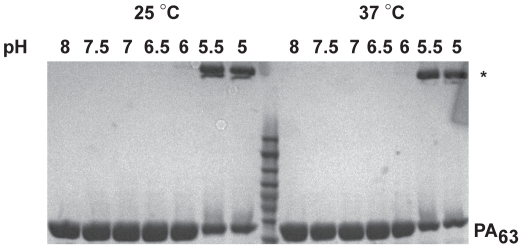
The formation of SDS-resistant PA_7_(LF_N_)_3_(msANTXR2)_7_ complexes is temperature-independent. SDS-resistance assays [25] were performed with PA_7_(LF_N_)_3_(msANTXR2)_7_ complexes, which were incubated at the indicated pH at either 25°C or 37°C. The two species of interest on the SDS-PAGE gels are indicated as either the high-molecular-weight, SDS-resistant PA oligomer band (*) or low-molecular-weight SDS-soluble, PA_63_ monomer band (PA_63_).

PA_7_ channel formation is both temperature-dependent as well as pH-dependent [22], [25]. However, while PA_7_ forms SDS-resistant aggregates upon channel formation, PA_8_ apparently does not [22]. We investigated the temperature-dependence of PA_7_-msANTXR2 channel formation using the SDS-resistance assay (Fig. 5). PA_7_-msANTXR2 complexes were incubated at 25°C or 37°C for 1 hour at pH conditions from pH 8.0 to pH 5.0. Using SDS-PAGE, we can monitor the formation of a low-mobility SDS-resistant form, which is likely an aggregated form of the heptameric PA channel [22]. In the absence of msANTXR2, PA_7_ forms an SDS-resistant species at pH 7.4 and 7.0, at 25°C and 37°C, respectively [22]. However, here we show, in the presence of msANTXR2, that the SDS-resistant species first appeared at pH 5.5 at 25°C and also at 37°C (Fig. 5). Therefore, we conclude that the pH-dependence of PA_7_-msANTXR2 channel formation is temperature-independent.

## Discussion

PA assembles on cell surfaces once it is proteolytically nicked by a cell-surface furin-type protease [33] after the RKKR sequence within a solvent accessible loop in domain 1 [20]. This solvent accessible loop is also recognized by an unknown serum protease found in various types of mammalian plasma [28]. The resulting products of the cleavage by either type of protease are the amino-terminal 20-kDa fragment, PA_20_, and the carboxy-terminal, 63-kDa fragment, PA_63_ [34]. The PA_63_ portion self-assembles into either a heptameric [17], [19], [20], [21] or octameric [21] ring-shaped oligomer, which can maximally bind up to three or four LF or EF molecules, respectively. Since assembly is initially limited by this proteolytic activation step, then Atx assembly can occur in two distinct environments, i.e., either (i) on cell surfaces or (ii) free in solution in extracellular environments such as plasma.

The cell-surface pathway is proposed to begin once secreted PA monomers bind to cell-surface ANTXRs [13], [14]. Furin-type proteases then activate PA, allowing toxin assembly and internalization to ensue. A complementary model has also been proposed based on predominantly *in vivo* results; the model suggests that PA monomers are proteolyzed in the host bloodstream [28], assembling into toxin complexes prior to reaching cell-surfaces [29]. We recently proposed that the octameric toxin stably circulates in plasma, while the heptameric toxin is unstable, forming aggregates of the prematurely formed channel state [21], [22]. Toxin aggregates have also been observed *in vivo*, although their oligomeric composition is unknown [28]. While the cell-surface assembly model and plasma-based assembly model are not mutually exclusive [21], [22], future *in vivo* studies are needed to distinguish the relative importance of the two assembly pathways during anthrax infection. Here we provide evidence that cell-surface assembly is likely driven by receptor dimerization; however, unique from plasma-based assembly, the two different oligomeric forms, PA_7_ and PA_8_, are similarly stabilized by ANTXR2 interactions on cell surfaces.

### ANTXR2 dimers stimulate PA oligomerization

The assembly of Atx on cell surfaces is not well understood. On one hand, proteolysis, assembly, and internalization have been shown to be rapid under physiological conditions, occurring within minutes [35]. The mechanism of cell-surface assembly is not fully understood in part because the structure of the full-length ANTXR is unknown and in part because individual assembly intermediates have not been isolated from cells. It is known, however, that while the soluble extracellular domain of ANTXR2 is monomeric, as evidenced in crystallographic [15], [17] and mass spectrometry studies (Fig. 2), the full-length ANTXR2 is thought to dimerize via its transmembrane single-pass helix domain, as demonstrated in studies of the transmembrane domain in a liposomal system [36]. Since ANTXR2 dimerization enhances the formation of PA_8_, it was proposed that it may also facilitate assembly at the cell-surface by populating dimeric intermediates, because PA_8_ complexes are produced in that environment [21]. In further support of this hypothesis, we also find evidence for even-numbered receptor-bound dimeric and tetrameric species, PA_2_(LF_N_)(msANTXR2)_2_ and PA_4_(LF_N_)_2_(msANTXR2)_4_, in our mass spectra (Fig. 2). We conclude that the cell-surface assembly mechanism likely occurs through dimeric PA intermediates, which are stabilized via dimeric ANTXR complexes; and this mechanism is akin to a putative extracellular plasma-based assembly mechanism, whereby either EF or LF stabilizes dimeric PA intermediates that can serve to drive assembly [21].

Previous studies have shown that the rate of PA assembly is accelerated in the presence of LF_N_ [16], which is believed to bridge a binding site spanning the surface of a PA dimer [37]. In this report, we consider the role of a dimeric receptor on the kinetics of PA assembly. Our kinetic FRET measurements show that dsANTXR2 stimulates PA assembly (Fig. 1B). Furthermore, PA assembles ∼6-fold faster with dsANTXR2 (relative to what is observed when assembling with LF_N_.) This acceleration of the assembly kinetics may reflect that PA binds ANTXR2 with a higher affinity than LF_N_ (170 pM versus 1 nM, respectively) [16], [38]. Therefore, we propose that ANTXRs, LF, and EF can stimulate PA oligomerization by populating dimeric PA intermediates, which are precursor intermediates in the PA oligomerization mechanism.

### Stabilization of PA complexes by ANTXR2

The ANTXR-dependent stabilization of PA oligomers has been demonstrated on cell-surfaces and in solution [17], [19], [25]. The mechanism of receptor-mediated stabilization of PA complexes occurs by reducing the pH threshold for PA channel formation by ∼2 pH units. ANTXR2 prevents channel formation by forming a metal-ion dependent structural bridge that spans domains 2 and 4, restricting the conformational changes necessary for channel formation [15], [17]. While PA_7_ complexes have been shown to be stabilized by ANTXR2 interactions [17], the relative stabilities of PA_7_ANTXR2_7_ and PA_8_ANTXR2_8_ complexes has not been reported. In the absence of msANTXR2, it has been demonstrated that PA_7_ and PA_8_ form channels at different pH values [22]. Our EM measurements of pH-dependent PA oligomer aggregation suggest that PA_7_- and PA_8_-msANTXR2 complexes instead form channels at equivalent pH values with a pH midpoint of 5.8 (Fig. 3B). Our CD measurements also suggest that PA_7_- and PA_8_-msANTXR2 complexes form channels at a pH midpoint of 5.8 (Fig. 4). Further evidence of this conclusion is provided by our analysis of the percentages of soluble PA_7_ and PA_8_ oligomer complexes over the tested pH range. From pH 8.0 to 6.0, we find the relative ratio of PA_7_ and PA_8_ complexes is unaltered, indicating that both PA_7_ and PA_8_ prechannels are stabilized by msANTXR2 (Fig. 3B). These results are consistent with studies of PA oligomerization on cell surfaces, revealing that the inherently less stable PA_7_ complex is favored 2∶1 over the PA_8_ complex [21]. Therefore, we infer that PA_7_ and PA_8_ are likely to form similar structural interactions with ANTXR2, since both oligomers are equally stabilized. We conclude that PA_7_- and PA_8_-msANTXR2 complexes form channels with identical pH dependencies and possess equivalent stability when bound to ANTXR2 on cell surfaces.

### Role of ANTXR2 stabilization during pathogenesis

**Figure 6:**
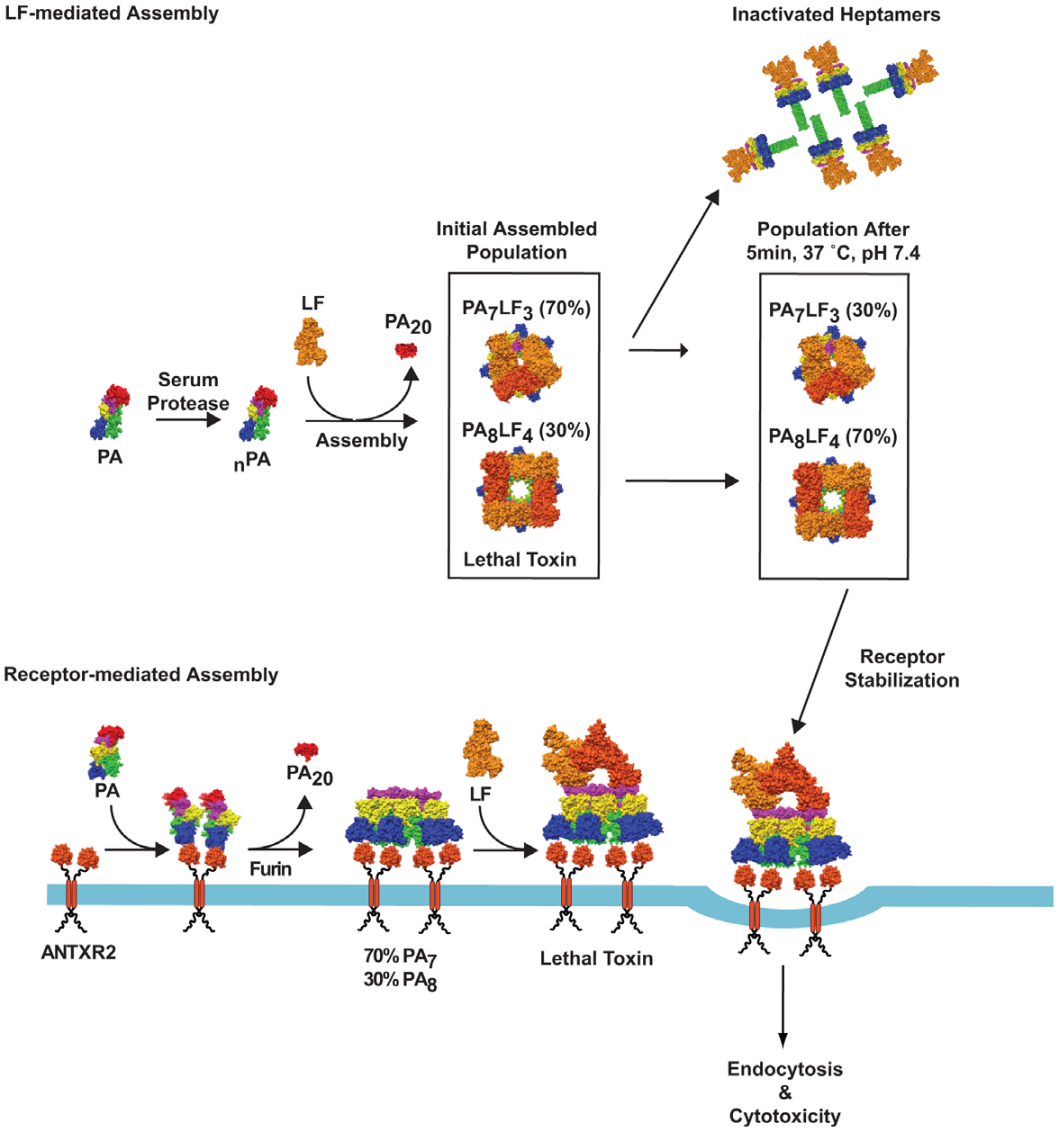
A model for anthrax toxin assembly. A model for anthrax toxin assembly in plasma and at cells surfaces. (**A**) In principle, PA components may assemble into a 70∶30 PA_7_:PA_8_ mixture of toxin complexes in plasma. However, PA_7_ readily converts to the channel state and aggregates within 5 minutes under these conditions, leaving PA_8_ as the predominant soluble toxin complex capable of infecting cells [22]. By contrast, both oligomeric forms are equally stable at the cell surface, where binding to ANTXR2 serves to prevent premature channel formation until PA_7_ or PA_8_ complexes are properly internalized and the endosomal compartment is acidified to pH values <6. On cell surfaces, PA may also oligomerize into a 70∶30 PA_7_:PA_8_ mixture [21], where assembly is driven through interactions with dimeric ANTXR complexes. These complexes are then able to bind LF and become internalized into cells.

**Figure 7:**
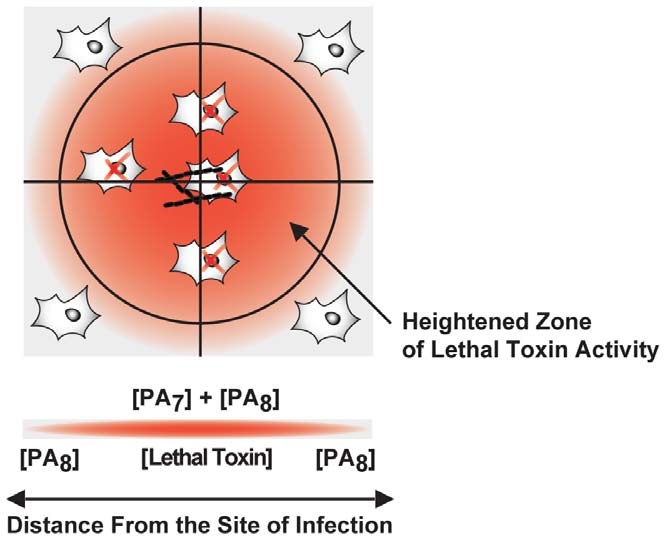
A model for the regulation of toxin activity in plasma. The assembly of LT complexes with different lifetimes may serve as a means to regulate toxin activity in plasma during infection. The reduced lifetime of PA_7_ complexes in plasma may limit their cytotoxic effects to local areas in close proximity to the site of *B. anthracis* infection. By contrast, PA_8_, which is produced at lower levels, has a longer lifetime, thereby allowing it to exert cytotoxic effects over longer distances.

In plasma, LT complexes containing PA_8_ oligomers are inherently more stable than those containing PA_7_ oligomers, thus allowing the LT containing PA_8_ complexes to persist for longer periods of time in that environment [22]. This stabilization mechanism defines an important role for PA_8_ oligomers in anthrax pathogenesis. However, as we report here, toxin complexes containing either PA_7_ or PA_8_ oligomers are equally stabilized by interactions with ANTXR2 (Fig. 6), and thus the 70∶30 ratio of heptamers to octamers observed on cell surfaces [21] can be fully explained by the receptor-mediated stabilization data presented here (Fig. 3B). Therefore, Atx assembly may be a means to regulate toxin activity and generate toxin gradients in the host (Fig. 7). We expect that, due to its relatively short half life, PA_7_ activity may effectively localize near to sites of infection [22], whereas PA_8_ may circulate systemically and provide a longer-range source of toxin activity. The stabilization imparted upon binding to the ANTXR allows for PA_7_ containing toxin complexes to be more concentrated and efficacious at sites proximal to the site of infection. The levels of available free LF may control the levels of PA_8_ complexes produced. This mechanism should provide a means to maintain higher levels of toxin activity near to the sites of infection while preventing premature system-wide shock until PA_8_ complexes are produced on a larger scale. As the infection progresses and a fever in the host develops, PA_8_ complexes may be required because they are more thermostable and remain active even after extended exposure to elevated temperatures [22]. Future work should investigate the types of Atx complexes produced throughout the various stages of anthrax pathogenesis.

## Materials and Methods

### Proteins

Recombinant wild-type PA_83_ [39] was over-expressed in the periplasm of the *Escherichia coli* strain, BL21(DE3). The 83-kDa PA monomer was purified from the periplasm as described [16]. Recombinant LF_N_ (LF residues 1-263) was overexpressed in BL21(DE3) via a pET15b construct [40] and then purified from the cytosol as described [16]. Soluble human anthrax receptor domain, msANTXR2, from the capillary morphogenesis protein 2 (residues 40–217) [14] was expressed and purified as described [15]. The six-histidine affinity tags were removed from msANTXR2 and LF_N_ by treatment with bovine α-thrombin. A soluble, dimeric fusion of human anthrax receptor domain to glutathione S-transferase (GST), dsANTXR2, was also expressed and purified as described [21].

### Preparation of purified PA_7_-msANTXR2 and PA_8_-msANTXR2 co-complexes

PA_7_(LF_N_)_3_ was produced using Q-sepharose-purified PA oligomers [21] by forming complexes with a two-fold stoichiometric excess of LF_N_ (LF_N_:PA) and purified as described [22]. The resulting complexes contained >90% PA_7_. PA_8_(LF_N_)_4_ was prepared by assembling _n_PA in the presence of LF_N_ (_n_PA-LF_N_, a mixture that contains ∼20–30% PA_8_), following incubation and purification by gel filtration as described [21], [22]. PA-msANTXR2 complexes were formed by mixing 1 µM PA_7_(LF_N_)_3_ or PA_8_(LF_N_)_4_ with ten molar equivalents of purified msANTXR2 (10 µM) in Buffer E (20 mM Tris, 150 mM NaCl, pH 8) plus 1 mM MgCl_2_. The complex was formed at room temperature over the course of 15 minutes.

### PA_83_ labeling with fluorescent dyes

A PA_83_ mutant K563C was expressed and purified in the presence of 5 mM DTT. Prior to the reaction, the DTT was removed by buffer exchange on a G25 desalting column (GE Healthcare, USA), equilibrated in nitrogen-purged Buffer E. Labeling reactions were initiated by mixing DTT-free PA_83_ K563C with 10 molar equivalents of Alexa fluor 555 C_5_ maleimide (AF_555_) or Alexa fluor 647 C_5_ maleimide (AF_647_) (Invitrogen, USA) in the presence of 100 µM tris(2-carboxyethyl)phosphine (TCEP, Sigma Aldrich, USA) and incubated at room temperature for 3 hours. The reaction was quenched with 5 mM DTT and purified on a G25 desalting column to remove free, unreacted dye molecules. Labeling efficiency was determined by comparing dye and protein absorbance values. Labeling efficiencies of >90% were achieved for either dye.

### FRET-based PA assembly assay

Dye-labeled, nicked PA (_n_PA K563C*AF_555_ or _n_PA K563C*AF_647_) was prepared as described previously [41]. To initiate assembly, _n_PA K563C*AF_555_ and _n_PA K563C*AF_647_ were each diluted to 50 nM in 10 mM sodium cacodylate, 100 mM potassium chloride, 1 mM magnesium chloride, pH 7.4 either in the presence or absence of 100 nM LF_N_, 100 nM msANTXR2, or 100 nM dsANTXR2. Assembly was observed as an increase in the emission intensity ratio at 668 and 566 (±2) nm (F_668_/F_566_) upon excitation at 555 (±5) nm, which reached a steady state in about one hour. Emission values were obtained every five minutes on a Horiba Jobin Yvon FluoroMax-3 spectrofluorometer, using quartz cuvettes with a 1-cm path length.

### Mass Spectrometry

Mass spectra of the protein complexes were acquired using a quadrupole time-of-flight (Q-TOF) mass spectrometer equipped with a Z-spray ion source (Q-TOF Premier, Waters, Milford, MA). Ions were formed using a nanoelectrospray (nano-ESI) emitter prepared by pulling borosilicate capillaries (1.0 mm O.D./0.78 mm I.D., Sutter Instruments, Novato CA) to a tip I.D. of ∼1 µm with a Flaming/Brown micropipette puller (Model P-87, Sutter). The instrument was calibrated with CsI clusters formed by nano-ESI using a 20 mg/mL solution of CsI in 70∶30 Milli-Q water:2-propanol prior to mass measurement. The protein solution was concentrated to ∼10 µm followed by dialysis into 10 mM ammonium bicarbonate, 1 mM magnesium acetate, pH 7.8. Immediately prior to mass analysis, the solution was diluted 1∶4 with 200 mM ammonium acetate, pH 7.8. A platinum wire (0.127 mm diameter, Sigma, St. Louis, MO) was inserted through the capillary into the solution and electrospray was initiated and maintained by applying 1–1.3 kV to the wire (relative to instrument ground). Each raw dataset was smoothed three times using the Waters MassLyn software mean smoothing algorithm with a window of 25 *m*/*z* (mass-charge ratio).

### Electron microscopy

PA-msANTXR2 complexes were prepared in Buffer E plus 1 mM MgCl_2_ as described above, applied to a freshly glow-discharged 400 mesh formvar-carbon coated grids, and stained with 2% uranyl acetate (Sigma-Aldrich, St. Louis, MO) as described [21], [22]. Negative-stain EM images were recorded on a Tecnai 12 electron microscope (FEI Company, Hillsboro, OR) operated at 120 kV at a magnification of 49,000× using a CCD camera. The micrograph resolution was 2.13 Å/pixel. Particle images were selected using manual particle picking using boxer in EMAN [42]. Boxed images of the PA oligomer particles were subjected to successive cycles of reference-free and reference-based alignment, multivariate statistical analysis, and classification using SPIDER [43], [44], [45], as described [21], [22]. Final class-average images were manually inspected to determine their oligomeric state and tabulated to determine the oligomeric composition of each sample (Table 2).

### Circular dichroism (CD) spectroscopy

CD measurements of the PA channel transition were obtained on a JASCO Model 810 spectropolarimeter (JASCO, Inc., Easton, MD). To determine the pH-dependence of the prechannel-to-channel transition, PA-msANTXR2 co-complexes were diluted to 50 nM in 2 mL of the buffer: 10 mM potassium phosphate, 10 mM potassium acetate, 0.1 M potassium chloride, 1 mM magnesium chloride, pH 8. The CD measurement was made using a 1×1-cm quartz cuvette containing a Teflon stir bar, at 25°C or 37°C. Recordings of the CD_222nm_ signal were conducted at a 1-Hz sampling rate. During the recording the pH of the sample was reduced by adding 0.4 M phosphoric acid to obtain the desired pH, as described [22]. The CD_222nm_ transition was then observed and recorded for an additional 60 s. The final pH of the sample in the cuvette was determined using a pH meter.

### SDS-resistance PAGE analysis

SDS-resistance assays [25] were performed with purified PA_7_-msANTXR2 complexes. The purified PA_7_(LF_N_)_3_ samples were complexed with msANTXR2 as described above, forming PA_7_-msANTXR2. PA_7_-msANTXR2 complexes were diluted to 1 mg/ml final concentration (with respect to PA) in Buffer E plus 1 mM MgCl_2_. The following buffers were added to preformed complexes, which vary depending upon the pH: 0.1 M Tris-Cl (pH 8.0), sodium cacodylate (pH 6.5 to 7.5), 0.1 M 2-(*N*-morpholino)ethanesulfonic acid (pH 6.0), and sodium acetate (pH 5.0 to 5.5). The complexes were incubated for 1 hour at 25°C or 37°C. 1.25% SDS then was added, and the samples were run on a 12% polyacrylamide gel, which was stained in Coomassie Brilliant Blue G-250.
